# Scirrhus of the Sub-Maxillary Gland

**Published:** 1853-01

**Authors:** 


### ARTICLE XX.

### *Scirrhus of the Sub-Maxillary Gland.*

Mr. Chilos reports the following case in the *London Lancet:*

Miss N——, aged thirty-five, of scorbutic habit, applied to me, March 8th, 1847, in consequence of a large tumor, situated beneath the left inferior maxilla, extending from the symphysis to the angle of the jaw. She had perceived it twelve years previously, when it appeared about the size of a small hazel nut; it had since been enlarging up to the present time; its shape was ovoid, and it felt pulpy to the touch, conveying to the finger the same sensation as is experienced on handling a fatty tumor. On making an exploratory incision with a small tendon-knife, a quantity of straw-colored fluid was ejected through the wound, and the tumor became much diminished in size; it was hence inferred that it was of a cystic character, and a pledget of lint was introduced through the opening, for the purpose of exciting inflammation, and if possible, obliteration of the sac. In about three days the lint came away, and the wound healed.

On examining the tumor, March 25th, although somewhat smaller, it was much firmer, and harder to the touch, and in accordance with the wishes of the patient, its removal was determined on. I was assisted by Mr. Gay, in the operation, which was tedious, on account of the intimate connections between the tumor and the surrounding tissues; and from the relations of the tumor with the vessels and nerves, each touch of the knife required the utmost caution. Five ligatures were applied; these were placed on the different vessels, previously to their division, by dissecting them carefully out, and passing a threaded aneurism-needle beneath. By the observance of this precaution, very little blood was lost. Ether had been inhaled, and the patient became insensible during the early stage of the operation. The lips of the wound were brought together, and retained in their situation by two sutures, covered by pledgets of lint dipped in cold water. This dressing remained on three days, and when removed, the wound being found rapidly cicatrizing, the sutures were taken away.

By the 7th of April two of the ligatures were detached, and the cicatrization was nearly complete. On the tenth day another ligature followed, and the parts appeared healthy. On the 14th, the dressings being removed, a dark spot, about the size of an issue-pea was seen, near the centre of the wound, immediately surrounding one of the remaining ligatures, and having the appearance of a small clot of venous blood beneath the cuticle; a quantity of dark, grumous discharge, mixed up with healthy pus, was deposited on the lint. The other portions of the wound looked healthy.

On April the 11th, seventeen days after the operation, the ligature, surrounded by the appearance alluded to, came away, and a small fungous-looking granulation occupied its place: this was freely cauterized with nitrate of silver, and dressed with dry lint; the patient complained of pain and uneasiness about the inferior maxilla, but there were no appearances of swelling, and with the exception of this fungous growth, the parts had assumed a healthy appearance.

April 14th.—The fungus was gone, and its situation occupied by a healthy cicatrizing granulation. The last ligature now came away, and the patient has remained well up to the present time.

*Examination of the tumor.*—After removal, it became much diminished in bulk, and very firm to the touch on cutting through it. Some parts presented an appearance similar to the carneæ columnæ of the ventricles of the heart. There were two cysts, each about the size of a walnut, situated near the circumference of the tumor, and filled by what appeared to be decomposed coagula and pus, and in character very similar to the softening of an encephaloid mass. These cysts were easily deprived of their contents, which were readily turned out with the finger. They were lined by a smooth membrane. Some portion of the gland appeared healthy.

